# Supplementary material for: Characterization of Trehalose-6-Phosphate Synthase and Trehalose-6-Phosphate Phosphatase Genes of Tomato (Solanum lycopersicum L.) and Analysis of Their Differential Expression in Response to Temperature
Source: Int J Mol Sci. 2022 Sep 28;23(19):11436. doi: 10.3390/ijms231911436 (PMC9569751; doi:10.3390/ijms231911436)
Supplement: Supplementary file 1 [file ijms-23-11436-s001.zip › ijms-1914717-supplementary.pdf]

Mollavali and Börnke; Supplementary Figure S1.

>pSlTPS2
GGTTGAGAACTGTCGAGATGTTAGCCGGACCAAAAAAGGAAATCAAGTAGACGTTTGAGTTTCCTTAG
CGCTCTTACGTTGACGGCCCAGTCCAATCCAACTCAGTTTTAGCGGGCTGGAAAGACTTTAGAAGGGC
CTCGAAAAGAGCATCTTACATAATTGTACAATTGATATACAATAATTATATAACTATATTTATTATAT
ATATGTATATACATATAAATATGTATTGTTTATATTTGAATAATACATGATACTTAACTTGGCATCCG
CAAATTCAATTTGAATGTGCACACACCCAAAATTAAAAATTAGAGTTGTCAACTGACATTAAGTTATT
ATGCCTTTATATTTCATAAAAGTTGTATATTTTAGATTGTGAATGCATGATGTAACTATTCATGTATT
TATGAAATGTAATATAGTTACTATATATATTAAGTGAGTAGATGAAAATAAGCTTTCATAATAATAGA
CGAAAAGATTGAAAAGCTAAACCGTAAGATACATAATCTATCAAACCTTTCAAGCGTATGAGATTCTT
GGGAAAATTTGTGCATTTTTATCCAAATTGAACAATATAATACATTGTTGAGAATATCTTTGAGCTAA
AAGAACACAATTACTAATATTAAAGTCTAGGTTTAACAGGAAGAATATGACGATGACAAATCATAGGA
CTCGACGAAAAAACTAATTGCAAACGTCTATTGCTAAAAGATACTCGAGTGATGTGGTTATGACACAA
TAAATTTTTAAAAAATCGACTCGTGGATTATGATAATGAGCCACATGAAACTTAGTTCAAAGGGGTGA
TTATTGTGTAGACAAACAGAATGTGACATTGAGTTAAAAGATTGAGAAACTATACTAAAATACACGAT
TTCTAAATGTTGTAAATTTTTTTATTTCAAAAAATATTTACGCTTTATCCAAAACAAACAATATCATA
CTTTATTAATAACGTGATTGCTCTCCTAACAAGAGTGAAAAAAAGGTTTACATTACCATTTAACACTT
TCATAATCACAATTTATACTACATTATTTTTTAGAAAATATGATTTTACTATATTTGAAAATATTTGT
GATTTTATTTTAAAGGTTTGTTGAGAGTTGAAGAGTTGAGTCCCTATGTGATTAAAAAAGAAGCTTTT
TGTGTAGTACCGTTGGGGTGCCAACAAAGGAGAAAAACAGGTGCGCATCTTGCATTGCATGGCGAAGA
AACCCGTGTTCCTCACAAACGCTTTCTTCTATCTTTTCACTCTCTTCAAAGGGTTTGGATTTTCTGTA
ATTTGACGAAACCCCTCTTTGTTCCTTTCCTCCATTTTTCTTTTCTGTAATTTTTCACATTTTTCCTC
CCTCCCTCCTTACATACGTACAAATTGTTGACAACTTGCTTCTCCTGTCTCTATATGGACTGCCTGCT
TTACACTTCTAAATCCCAATTTCTCATTTGGGTCTTTATTATTATTATTCTTATTGGGATTGACGCTG
CTGAAG**ATG**

>pSlTPS8
CCTATTATATCCTCAATTAATTATTTTAAAAAGGTAACTTTTTTTTTTGTTCTTATTAATAGATGTAT
CAATTCAATAATGGACAAGTAGATTTTCATAACTAAAATATGAGTATCTCTTGAGGCGTCGTATTAAT
AAATCATGTATCTTCAAATTTCTTTTAATAAATAATTTTATAAAGATTCAATAATAGTAATAACTAAT
TATTCTTTAATATAATTTTTTTATTTTTTTATTTAAGTTTTGTAAAATTTAAAAGTATAAATCTTTCT
TTTTACAACATATAAATTTATGATGAAGCTTTACATTTTTATACGGCTGATTATCTACTTTGTTTATT
AAAGGTTTATCCCAAAATAATAAAATAACAAAAAATATAACATTAATCAAAATTAAAATTAATGATAA
TGATGGAGAATTAAAATTCGAGGTGGGCTAGATAGATGAGTAATTTTAGATTAATTCCAGGAATGTGC
AGTCCGCGCAGCCTTTGAGGCCTTTTAAAAATAGGAATCTGAAAAAATAAAAAATCTGTGTGTCAGCT
GCATGGCTGTAAGGCTTTATGCACCTTTTAAAGCTTACACGAGGATTCGTCTGCTCTTCCACTTCCCC
TCTTTAGCTATGATAATAATTCCTCTGCCACCCGGTATTATCGATTTGCTCTCACAAATTCATCATCA
AATGCTACTAATTTTTAATAATTACTCATAATTATTGTTGTGATCAAGCTTTTCCGGCCGGTTATCGA
TGTTGAGATCAGATTAGGCCAGGAATCCTTCCCCTTGTTTTTCACATCGGTTTTACAGGTAATTTGAC
ACAGTCTGGATTTGATTTACCGTGGTTTCTGAAATGGGTTAATTAGCTGTCACAGAAATCTCTGTGCC
TGTGTTCTTTTACAGCTTTCTCAATGAATTCGGGGTTTGGTTTTCTGCATGCAATGTGTTTGTGCCGG
TGTCCGCTTTGAATCTGATAGCTGTTCTTTTTAATCTGGTGGGTCTTTATATGTTCACACTTGCCTCA
TTGTATTTTGCTTGAACTGAAACTGGGATACCAAATGAATTTATATTTAAAATCCTGGACAGAAAAAG
CCAAGACTCTTGGGGTGGTAGACCCCATTTCCCTAGAGAATTCTGTGGATCCCATTCTCCACACGTTA
TCTCTTTCTTAGTTTAAATCCTTTTTCTCTCTAGTACTACTGATTTTCAATAATTTTAAGTATTGGAT
TTATATGGGTCTAAATGGAGTGACATGGTTAGTGAAGTGGTGTTATTGTTGTAGTGGTTTATTCAACT
AGGATGCTGATGTCCTATTCATTTATGTTAGCCTTGGCTGTGAAATTGCATTTTCACTGTTTGTACTT
GAGGCTATTGTGGAATTGTATAAGTTTTTATATAATTCACGCGGGAGTTTCATCTTTTGTTACCTCTT
ATTATGAAAGATGGTTGTTTCCAGGGACATCATAACTCTATTAAATTCATCATAGGTGGTCTTGTTCT
AGACAG**ATG**

Supplementary Figure S1. *SlTPS2* and *SlTPS8* promoter sequences 1.5. kb upstream of the translational start site (bold).

Mollavali and Börnke; Supplementary Table S1.

Table S1. ***TPS/TPP*-genes in tomato (*Solanum lycopersicum*)**

Family	Gene	Locus ID	GenBank Acc. No.	ORF (bp)	Protein MW KDa	PCR
TPS	*SlTPS1*	Solyc01g005210	XP_004228746	2574	97	+
	SlTPS2	Solyc02g071590	XP_010316884	2832	106	+
	SlTPS3	Solyc02g072150	XP_004233035	2556	96	+
	SlTPS4	Solyc04g025940	XP_004237260	2574	96	+
	SlTPS5	Solyc05g005750	XP_004238680	2556	96	+
	SlTPS6	Solyc07g006500	XP_010323144	2631	96	+
	SlTPS7	Solyc07g055300	XP_004243268	2577	97	+
	SlTPS8	Solyc07g062140	NP_001234879	2781	104	+
	SlTPS9	Solyc08g076650	XP_004245918	2589	97	+
	SlTPS10	Solyc10g007950	XP_004248198	2574	97	+
	*SlTPS11**	Solyc10g046770	XM_010329326	735	28	-
TPP	*SlTPP1*	Solyc03g007290	XP_004234173	1011	39	+
	SlTPP2	Solyc03g083960	XP_025885725.1	1154	43	+
	SlTPP3	Solyc04g054930	XP_004237406	1167	44	+
	SlTPP4	Solyc04g072920	XP_004237894	1098	41	+
	SlTPP5	Solyc04g082550	XP_025886308	882	33	-
	SlTPP6	Solyc05g051880	XP_019069377.1	905	34	-
	SlTPP7	Solyc06g060600	XP_004242008	1020	38	-
	SlTPP8	Solyc08g079060	XP_004245739	1161	43	+

Mollavali and Börnke; Supplementary Table 2.

Supplementary Table 2. Nucleotide sequences of gene-specific primers.		
**Target gene**		**Primer sequences used for qRT-PCR primers**
SlTPS1	Fw	5'-GCTCCAGCAGCCGAAGTCTT-3'
	Rv	5'-CCTGCATCAGCCTAACTATCTC-3'
SlTPS2	Fw	5'-TCTTTTCCATTATCTCCCACTTCCG -3'
	Rv	5'- GCTGTCCTTGGTCCTCTACTCCTTC-3'
SlTPS3	Fw	5'- CTTCCAGTGAGAGAGGAGAT-3'
	Rv	5'- CATGCGACTACAACAGGATAG-3'
SlTPS4	Fw	5'- AGAAGCCAAGCAAAGCCAAG-3'
	Rv	5'- AGAAGCATTAGCAAGACCTCCA-3'
SlTPS5	Fw	5'- AAAGCCAAGCAAAGCCAAGTA-3
	Rv	5'- GACAGGACGCATTACCAAGAC-3'
SlTPS6	Fw	5'- TTCAGCAGCTTCTACAGCAATG-3'
	Rv	5'- CTAGGGAGAGATCTTCGACAGA-3'
SlTPS7	Fw	5'- ACACATCTGAGGTGGTGCTTAT-3'
	Rv	5'- GGAGTCCGCAGGTTCATCGT-3
SlTPS8	Fw	5'- GTGTCATGGAATGTGCTGGAT-3'
	Rv	5'- TGGCGTACTGAGCAGATACC-3
SlTPS9	Fw	5'- GGCCAGAAACCAAGCAAGG-3'
	Rv	5'- CTCAGAAGCAGAAGCCAGG-3'
SlTPS10	Fw	5'- TCGGTGATGCTTTATCCAGAAA-3'
	Rv	5'- AACCTCTGAAGTGTCGTCCAA-3'
SlTPS11	Fw	5'- TGTGGTTTACAACAATAGGGTGG-3'
	Rv	5'- TGTCTAGAATCGATTATGTCCGG-3'
SlTPP1	Fw	5'- TGAACGTGCCTTCATGTC-3'
	Rv	5'- CTGTCGACTTCTTCCACTTATT-3'
SlTPP2	Fw	5'- AGGAGATGATCGTACAGATGAA-3'
	Rv	5'- GATCCCTCAGAGAGTAGAATGC-3'
SlTPP3	Fw	5'- CTGAGGTTATGGTGTTTCTACG-3'
	Rv	5'- GTTCCTTAGAGATGCTTTCATC-3'
SlTPP4	Fw	5'- AGGCTTTGGAATATTAGTGTCC-3'
	Rv	5'- CCACTCCACCAAACGATTCAAA-3'
SlTPP5	Fw	5'- GAGAATGGTGCTAGGTGAAG-3'
	Rv	5'- CGATTTCCTACAGCTATTATCAGTGGACGA-3'
SlTPP6	Fw	5'- GACTATGATGGTACCCTTTCTC-3'
	Rv	5'- TGCACCTCCCACTAACTAT-3'
SlTPP7	Fw	5'- CGTGATAGACTGGGACAAAG-3'
	Rv	5'- CTGTTCTGTCATCGCCAATA-3'
SlTPP8	Fw	5'- GTGGGAGAAGACGTGATAAG-3'
	Rv	5'- GCAGACGATGTATTCTTGATTG-3'
SlTRE1	Fw	5'- ACGGGTTTCGGATGGTCAAA-3'
	Rv	5'- GCAGTCATTCTTCAAATCTTCG-3'
Ubiquitin	Fw	5'- TCGTAAGGAGTGCCCTAATGCTGA-3'
	Rv	5'- CAATCGCCTCCAGCCTTGTTGTAA-3'
		**Primer sequences used for cloning**
SlTPS1	Fw	5'-GTCGACACAAATGGTGTCAAGATCCTATTC-3'
	Rv	5'-TCTAGACATCTGTGGTAACATTTGGTC-3'
SlTPS2	Fw	5'-GGATCCACAAATGGAGTCGATTGAAGATAC-3'
	Rv	5'-AAGCTTGCAAGATGAATTATTGGCC-3'

SlTPS3	Fw	5'-GTCGACACAAATGATGTCCAAATCTTATAC-3'
	Rv	5'-TCTAGATGTTACAATTTCTGATGGAGG-3'
SlTPS4	Fw	5'-GGATCCACAAATGGCATCAAGATCTAG-3'
	Rv	5'-CTCGAGAATAACAGAACCAAAGGC-3'
SlTPS5	Fw	5'-GGATCCACAAATGCCATCAAGATCTTG-3'
	Rv	5'-CTCGAGCAGAACAGAGTCGAATGC-3'
SlTPS6	Fw	5'-GGATCCACAAATGTTGATGAATGTTCCAGG-3'
	Rv	5'-GTCGACTACAAAGGATGCATGATTC-3'
SlTPS7	Fw	5'-GGATCCACAAATGATGTCTAGATCGTATAC-3'
	Rv	5'-CTCGAGGTCGGAGTCCGCAGGTTC-3'
SlTPS8	Fw	5'-GGATCCACAAATGCCAGGGAACAAGTATAC-3'
	Rv	5'-GTCGACTGATGTCCCATTTGAAATAG-3'
SlTPS9	Fw	5'-GTCGACACAAATGGTTTCAAGGTCATATTC-3'
	Rv	5'-CTCGAGTTCTCGGTCAATGATTACGC-3'
SlTPS10	Fw	5'-GGATCCACAAATGATTTCGAGATCGTATAC-3'
	Rv	5'-CTCGAGAGCCGAGTCCTCAGCTTC-3'
SlTPP1	Fw	5'-GGATCCACAAATGAGAGAAAATTTGACAAG-3'
	Rv	5'-GAATTCCACCGCGGAAACCTCCCC-3'
SlTPP2	Fw	5'-AAGCTTACAAATGGACCTGAAATCAAATAC-3'
	Rv	5'-GTCGACTACCTCCGATGGATCCCTC-3'
SlTPP3	Fw	5'-GATATCACAAATGACTCAGCAGAATG-3'
	Rv	5'-CTCGAGGTTCCTTAGAGATGCTTTC-3'
SlTPP4	Fw	5'-GGATCCACAAATGACTAATCAGAATG-3'
	Rv	5'-AAGCTTTTTCTTCTGATATTTTTG-3'
SlTPP8	Fw	5'-GGATCCACAAATGGACTTGAATTCG-3'
	Rv	5'-GATATCTATATCATCATCTTCAAC-3'
		**Primer sequences used for construction of *GUS::ProSlTPS2,8***
ProSlTPS2::GUS	Fw	5'-GGTTGAGAACTGTCGAGATGTTAGC-3'
	Rv	5'- CTTCAGCAGCGTCAATCCC-3'
ProSlTPS8::GUS	Fw	5'- GATGTATCAATTCAATAATGGAC-3'
	Rv	3'- CTAGAACAAGACCACCTATGATG-3'
